# Supplementary material for: FHBF: Federated hybrid boosted forests with dropout rates for supervised learning tasks across highly imbalanced clinical datasets
Source: Patterns (N Y). 2024 Jan 12;5(1):100893. doi: 10.1016/j.patter.2023.100893 (PMC10801222; doi:10.1016/j.patter.2023.100893)
Supplement: Document S1. Table S1 [file mmc1.pdf]

Patterns, Volume *5*

Supplemental information

FHBF: Federated hybrid boosted forests with dropout rates for supervised learning tasks across highly imbalanced clinical datasets

Vasileios C. Pezoulas, Fanis Kalatzis, Themis P. Exarchos, Andreas Goules, Athanasios G. Tzioufas, and Dimitrios I. Fotiadis

Supplementary Material

Supplementary Table 1. Descriptive statistics of the features participating in cases 1-8.

ID	Feature	Type	Range	Mean/Most frequent value
1	Gender	discrete	[0, 1]	1 (females)
2	Age of SS diagnosis	continuous	[10, 95]	51.59
3	Disease duration	continuous	[0, 49]	7.21
4	Dry mouth	discrete	[0, 1]	1 (presence)
5	Dry eyes	discrete	[0, 1]	1 (presence)
6	Parotid or Submandibular swelling	discrete	[0, 1]	0 (absence)
7	Raynaud"s phenomenon	discrete	[0, 1]	0 (absence)
8	Arthritis	discrete	[0, 1]	0 (absence)
9	Renal disease	discrete	[0, 1]	0 (absence)
10	Tubulointerstitial nephritis	discrete	[0, 1]	0 (absence)
11	Glomerulopathy	discrete	[0, 1]	0 (absence)
12	Pulmonary disease	discrete	[0, 1]	0 (absence)
13	Small airway disease	discrete	[0, 1]	0 (absence)
14	Interstitial lung disease	discrete	[0, 1]	0 (absence)
15	Liver disease	discrete	[0, 1]	0 (absence)
16	Autoimmune hepatitis (AIH)	discrete	[0, 1]	0 (absence)
17	Primary biliary cholangitis (PBC)	discrete	[0, 1]	0 (absence)

18	Nervous System Disease	discrete	[0, 1]	0 (absence)
19	Peripheral Nervous System Disease	discrete	[0, 1]	0 (absence)
20	Central Nervous System Disease	discrete	[0, 1]	0 (absence)
21	Palpable purpura	discrete	[0, 1]	0 (absence)
22	Muscular System Disease	discrete	[0, 1]	0 (absence)
23	Idiopathic Inflammatory Myopathy (IIM)	discrete	[0, 1]	0 (absence)
24	Inclusion Body Myositis (IBM) documented with Biopsy	discrete	[0, 1]	0 (absence)
25	Anti-La-SSB	discrete	[0, 1]	0 (absence)
26	Anti-Ro-SSA	discrete	[0, 1]	1 (presence)
27	Rheumatoid Factor (RF)	discrete	[0, 1]	1 (presence)
28	Antinuclear Antibodies (ANA)	discrete	[0, 1]	1 (presence)
29	Low C4	discrete	[0, 1]	0 (absence)
30	Cryoglobulinemia	discrete	[0, 1]	0 (absence)
31	Lymphoma	discrete	[0, 1]	0 (absence)
32	B-cell Mucosa-associated Lymphoid Tissue (MALT) Lymphoma	discrete	[0, 1]	0 (absence)
