# Supplementary material for: FHBF: Federated hybrid boosted forests with dropout rates for supervised learning tasks across highly imbalanced clinical datasets
Source: Patterns (N Y). 2024 Jan 12;5(1):100893. doi: 10.1016/j.patter.2023.100893 (PMC10801222; doi:10.1016/j.patter.2023.100893)
Supplement: Document S2. Article plus supplemental information [file mmc2.pdf]

Article

Patterns

FHBF: Federated hybrid boosted forests with dropout rates for supervised learning tasks across highly imbalanced clinical datasets

Highlights

- Significant overfitting effects are introduced during federated learning
- Existing federated learning algorithms suffer from such ill-posed effects
- We propose a loss function that is resilient against overfitting effects
- The proposed hybrid booster is explainable and robust for decision making

Authors

Vasileios C. Pezoulas, Fanis Kalatzis, Themis P. Exarchos, Andreas Goules, Athanasios G. Tzioufas, Dimitrios I. Fotiadis

Correspondence

fotiadis@uoi.gr

In brief

In this work, the authors present a new algorithm for intensive federated learning tasks across highly imbalanced medical databases within the cloud. They refer to this algorithm as federated hybrid boosted forests (FHBF). FHBF is an explainable AI algorithm that uses a hybrid loss function to overcome ill-posed problems during training. They evaluate the proposed algorithm across a large pan-European data hub, where FHBF achieved the lowest average loss and best classification performance against existing federated learning implementations.

Pezoulas et al., 2024, Patterns 5, 100893
January 12, 2024 © 2023 The Author(s).
https://doi.org/10.1016/j.patter.2023.100893

CellPress

Patterns

CellPress
OPEN ACCESS

Article

FHBF: Federated hybrid boosted forests with dropout rates for supervised learning tasks across highly imbalanced clinical datasets

Vasileios C. Pezoulas,[1] Fanis Kalatzis,[1] Themis P. Exarchos,[1,2] Andreas Goules,[3] Athanasios G. Tzioufas,[3] and Dimitrios I. Fotiadis[1,4,5,*]

[1]Unit of Medical Technology and Intelligent Information Systems, Department of Materials Science and Engineering, University of Ioannina, 45110 Ioannina, Greece

[2]Department of Informatics, Ionian University, 49100 Corfu, Greece

[3]Department of Pathophysiology, Faculty of Medicine, National and Kapodistrian University of Athens (NKUA), 15772 Athens, Greece

[4]Biomedical Research Institute, FORTH, 45110 Ioannina, Greece

[5]Lead contact

*Correspondence: fotiadis@uoi.gr

https://doi.org/10.1016/j.patter.2023.100893

THE BIGGER PICTURE Federated learning (FL) is a decentralized strategy to train machine learning models that involves the cooperation of several sources. Through this approach, each source makes use of its own data for model training, which ultimately decreases the chances for data breaches and the use of high computational resources. In the end, the trained models, not the data, are shared between sources for updates over multiple iterations. These approaches are particularly important in research fields where the data include sensitive information, such as medicine, where clinical data involve details about patients, which are subjected to legal and ethical regulations. Despite its many advantages, however, training a model in a federated environment does not come without issues. One problem, for example, is the differences between datasets, which is the quantity and distribution of data. These imbalanced datasets could result in a performance decrease in the common model. Therefore, developing strategies to mitigate the impact of imbalanced data sets in FL is important to guarantee that a model is properly trained and accurate while still retaining the greatest advantage of FL: enhanced data privacy.

1 2 3 4 5 **Concept:** Basic principles of a new data science output observed and reported

SUMMARY

Although several studies have deployed gradient boosting trees (GBT) as a robust classifier for federated learning tasks (federated GBT [FGBT]), even with dropout rates (federated gradient boosting trees with dropout rate [FDART]), none of them have investigated the overfitting effects of FGBT across heterogeneous and highly imbalanced datasets within federated environments nor the effect of dropouts in the loss function. In this work, we present the federated hybrid boosted forests (FHBF) algorithm, which incorporates a hybrid weight update approach to overcome ill-posed problems that arise from overfitting effects during the training across highly imbalanced datasets in the cloud. Eight case studies were conducted to stress the performance of FHBF against existing algorithms toward the development of robust AI models for lymphoma development across 18 European federated databases. Our results highlight the robustness of FHBF, yielding an average loss of 0.527 compared with FGBT (0.611) and FDART (0.584) with increased classification performance (0.938 sensitivity, 0.732 specificity).

This is an open access article under the CC BY license (http://creativecommons.org/licenses/by/4.0/).

INTRODUCTION

Big data in healthcare can provide broader and more comprehensive insights on the optimization of existing healthcare services to leverage the financial burden of unnecessary patient readmission, to enable cost effective treatment, and to improve the patient's quality of life (QoL).[1–3] There is no doubt that the sharing of diverse clinical data from multiple data sources can promote research in healthcare. Toward this direction, the conventional strategy for knowledge mining across complex big datasets from multiple data sources is based on the co-analysis of the shared data,[4] which is usually referred to as centralized analysis. This type of analysis, however, is not always feasible due to GDPR (General Data Protection Regulation) violations and increased risk for data breach, as well as, due to the increased computational complexity, which is introduced during the training of machine learning (ML) or deep learning workflows across complex data.[5] A solution to this critical issue is to utilize a federated data management strategy,[6,7] where the diverse medical data from multiple sources are shared and stored under federated databases. In this case, the federated learning process is orchestrated by a central node that communicates with each individual node for the training process (i.e., for the ML model's weight update process).

A technical, as well as a legal and an ethical challenge in federated data management systems, however, lies on the training of robust federated ML workflows across diverse data that are stored in federated databases.[8] Toward this direction, batch processing methods such as online learning and meta-learning[9,10] have been proposed in the literature, where the former[9] uses stochastic optimization to update an existing estimator on upcoming training batches, whereas the latter[10] focuses on the aggregation of outcomes from models that are trained on each federated database. Meta-learning methods, however, limit the "horizon" of the training process since the individual ML models are trained on individual subsets.[2] Online learning methods, on the other hand, are restricted to the additive update of the weights of the ML model on new "online" training instances. A solution to this is to use incremental learning,[11–14] which trains a classifier on an initial database and then incrementally adjusts the weights of the classifier on a series of existing databases. Toward this direction, many incremental learning algorithms have been proposed, including the family of the multiple additive regression trees (MART),[14–16] the support vector machines (SVM),[17,18] and the multinomial naive Bayes,[14] where the gradient boosting trees (GBT) algorithm is the most popular implementation of MART with favorable performance in diverse classification tasks.[14,15]

A common problem with the FGBT algorithm, however, lies in the fact that the trees that are added early in the ensemble, at a particular stage, tend to have a higher impact during the decision-making process than those added later.[14,20] To this end, dropouts have been recently adopted by the deep learning community[19,20] to deal with this issue, by scaling the most prominent trees in the ensemble, with a specific rate of rejected trees. On the other hand, a main problem in FGBT with dropouts is to account for overfitting effects in the selection of the dropout rate which is arbitrary. Besides, the data inconsistency in each federated database combined with increased class imbalance can affect the weight update process yielding zero or infinite weights. In addition, federated random forests (FRF) models[21] have been proposed to overcome biases arising from the heterogeneity within and between datasets. On the other hand, even though federated implementations of conventional supervised learning algorithms, like the SVMs and logistic regression, are easy to be implemented and deployed in federated environments they are often prone to overfitting effects since they suffer by linearity assumptions and thus fail to capture complex data structures. On the other hand, naive Bayes approaches, like the multinomial naive Bayes, are partially affected by overfitting, are often less flexible since they assume feature independence. In addition, although the GBT (with and without dropouts) algorithm has been widely used in the literature as a state-of-the art classifier, with advanced implementations both in centralized and federated environments,[14–16,19] none of these existing studies have investigated the loss during the training and testing across multiple and highly imbalanced datasets within federated environments.

In this work, we propose the federated hybrid boosted forests (FHBF) algorithm, which implements a hybrid weight update process to deal with ill-posed problems that arise from overfitting effects during the training across complex and highly imbalanced data that are stored in federated databases. A scaling parameter is introduced to control the shape of the hybrid loss function based on the dropout rate to avoid overfitting effects. The FHBF currently supports both the hybrid FGBT (HFGBT) and the hybrid federated gradient boosting trees with dropout rate (FDART) (HFDART) as boosters. Class imbalance handling functionalities are incorporated to develop clusters of HFDARTs, where each cluster is formulated based on a random subset of the federated training instances. Then, a log loss score is used to isolate the weak sets of regression trees for the classification task under investigation to further boost the classification performance of the algorithm and increase its resilience against weak decisions. Shapley additive explanations (SHAP) analysis is applied as a final step to yield explainable outcomes. Eight case studies were conducted to demonstrate the superiority of the FHBF to solve demanding classifications tasks involving 18 federated databases against existing federated learning implementation. Our results highlight the robustness and resilience of the FHBF against overfitting effects during training and testing, yielding an average loss 0.527 across the eight cases compared with the FGBT (0.611) and the FDART (0.65 for *rd* 0.1, 0.582 for *rd* 0.2) along with increased classification performance, which reached 0.938 sensitivity and 0.732 specificity, supporting explainable outcomes based on the HFGBT booster.

RESULTS

Experimental phases

To evaluate the performance of the FHBF against the existing high-performance federated learning algorithms (i.e., FGBT and FDART[14]), we gained access to a pan-European data hub on rare autoimmune diseases, which includes 21 databases as part of the HarmonicSS EU Project.[31] The patient data were shared, curated, harmonized,[14] and stored in private spaces within a cloud environment. Socio-demographic

CellPress
OPEN ACCESS

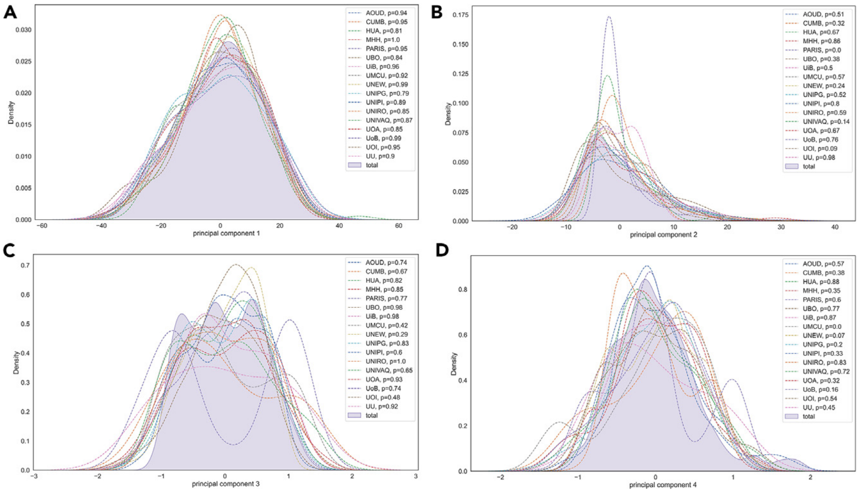

Figure 1. Distribution of the first four principal components (PCs)

(A–D) per federated database along with the first four incremental PCs across all databases (shaded area).

(A) Distribution of IPC1 versus PC1 from each database, (B) Distribution of IPC2 versus PC2 from each database, (C) Distribution of IPC3 versus PC3 from each database, (D) Distribution of IPC4 versus PC4 from each database.Figure 4. Distribution of IPC1 versus PC1 from each database.

information was present for 6,060 patient records that were included in the analysis. Different training and testing sequences were evaluated, involving either two or more databases for the training process and either one or more cohorts for the validation process. Two experimental phases were defined to examine the behavior of the FHBF algorithm across highly imbalanced data. To do so, the target feature in experimental design phase 1 was set to lymphoma, which has a 5% occurrence in the primary Sjögren's syndrome (pSS) population. The final number of eligible databases was reduced to 18 databases, since 5 databases had no reported lymphoma patients and thus were discarded from the analysis. The final number of harmonized patients was reduced to 4,905 with 32 overlapping features (Table S1). In experimental design phase 2, the classification problem was more difficult having even lower class imbalance, where the target feature was set to MALT (mucosa-associated lymphoid tissue) lymphoma, which is a lymphoma subtype with occurrence less than 3% in the pSS population. In this case, the final number of eligible databases was 17 (databases with no MALT patients were excluded from the analysis) with 4,805 patients.

Eight case studies were defined in experimental phases 1 and 2 with random training order and different testing databases to extensively evaluate the classification performance and the average training loss of the FHBF compared with the FGBT and FDART implementations. In the first experimental phase: (1) case 1 involves the federated training across 18 databases (“UOA,” “UNIPI,” “UNEW,” “UNIPG,” “PARIS,” “UoB,” “UNIVAQ,” “HUA,” “UOI,” “UU,” “UNIRO,” “UMCU,” “MHH,” “UBO”) and testing in a single database (“AOUD”), (2) case 2 involves the federated training in a different combination (“UOA,” “UNIPI,” “UNEW,” “UNIPG,” “PARIS,” “UoB,” “UNIVAQ,” “HUA,” “UOI,” “UU,” “UNIRO,” “UMCU,” “MHH,” “UBO”) and testing in the same database as in case 1, (3) case 3 involves the training across 18 databases (“AOUD,” “UOA,” “UNIPI,” “UNEW,” “UNIPG,” “PARIS,” “UoB,” “UNIVAQ,” “UOI,” “UU,” “UNIRO,” “UMCU,” “MHH,” “UBO”) and testing in a different database than in cases 1 and 2 (“HUA”), and (4) case 4 involves the training across 18 databases (“AOUD,” “UOA,” “UNIPI,” “UNEW,” “PARIS,” “UoB,” “UNIVAQ,” “HUA,” “UOI,” “UNIRO,” “UMCU,” “MHH,” “UBO,” “UU”) and testing in a different database than cases 1, 2, and 3 (“UNIPG”).

In the second experimental phase: (1) case 5 involves the federated training across 18 databases (“UOA,” “UNIPI,” “UNEW,” “UNIPG,” “PARIS,” “UoB,” “UNIVAQ,” “HUA,” “UOI,” “UU,” “UNIRO,” “UMCU,” “UBO”) and testing in a single database (“AOUD”), (2) case 6 involves the federated training in a different combination (“AOUD,” “UOA,” “UNIPI,” “UNEW,” “UNIPG,” “PARIS,” “UoB,” “UNIVAQ,” “HUA,” “UOI,” “UNIRO,” “UMCU,” “UBO”) and testing in a different database (“UU”), (3) case 7 involves the training across 18 databases (“AOUD,” “UOA,” “UNIPI,” “UNEW,” “PARIS,” “UoB,” “UNIVAQ,” “HUA,” “UOI,” “UNIRO,” “UMCU,” “UBO,” “UU”) and testing in a different database (“UNIPG”), and (4) case 8 involves the training across 18 databases (“UOA,” “AOUD,” “UNIPI,” “UNEW,” “UNIPG,” “PARIS,” “UoB,” “UNIVAQ,” “UOI,” “UNIRO,” “UMCU,” “UBO,” “UU”) and testing in a different database than cases 5, 6, and 7 (“HUA”).

Federated data consistency

The consistency of each harmonized database was first evaluated prior to the application of the FHBF to avoid biases during the incremental weight update process. To this end, principal-component analysis (PCA) was applied on each individual harmonized database to extract the first four principal components (PCs) as those that describe most of the variance in each database.

To compare the consistency of the four PCs from each individual database with the PCs across the total databases, we extended the PCA algorithm, where an empty PCA object was first fitted on a federated database to yield a low rank approximation, which was incrementally adjusted on the rest of the databases yielding incremental PCs (IPCs) 1–4.[32] IPCs 1–4 were compared against PCs 1–4 from each individual database using either the Student's t test or the Wilcoxon rank-sum test based on the normality estimations that were obtained by the Shapiro-Wilk test for normality. According to Figure 1, no significant differences were observed between IPC1 and PC1 per database, which confirms the consistency of the harmonized data. The same stands for IPC3 and PC3. Only one statistically significant difference was observed between PC2 and IPC2 and between IPC4 and PC4 in the PARIS ($p < 0.05$) and UMCU ($p < 0.05$) databases, respectively.

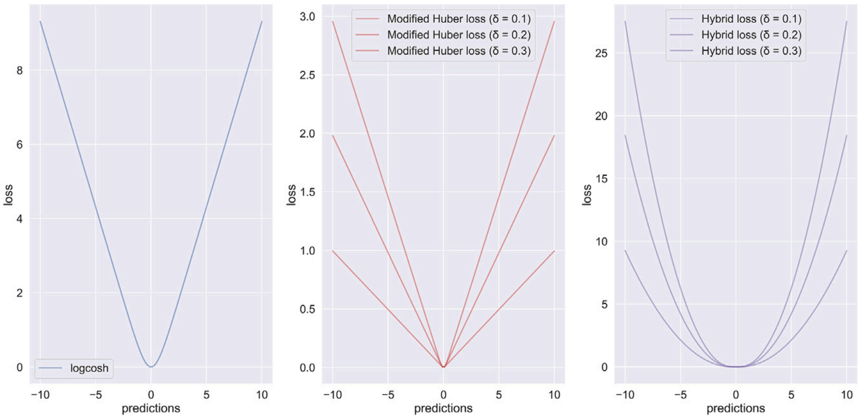

Figure 2. Loss function topologies
Topology of (A) the logcosh loss, (B) the modified Huber loss for δ values in the range 0.1–0.3, and (C) the proposed hybrid loss function for δ values in the same value range.

Benchmarking results from the experimental phases

The topology of the proposed hybrid loss function for different δ values (i.e., $\delta \in [0.1, 0.3]$) is depicted in Figure 2. For comparison purposes, the topologies of the logcosh loss and the modified Huber loss (for the same δ values) are also presented. For demonstration purposes, the horizontal axis was set to the range (−10, 10). According to Figure 1, the hybrid loss function combines the steepness of the modified Huber loss (Figure 1A) and the wideness of the logcosh loss (Figure 1B) into a new loss with a smoother topology (Figure 1C), where the scale of the topology is controlled by the δ value to control for overfitting effects. Since the δ value is directly linked to the dropout rate, larger dropout rates lead to a steeper loss topology, thus yielding higher penalties during the weight update function.

The FHBF, FGBT, and FDART with dropout rates 0.1 and 0.2 were used to solve a sequence of intensive supervised learning problems across the eight cases from experimental phases 1 and 2. In the FGBT algorithm, the booster was set to the "gbtree," the objective to "binary:logistic" and the "eval_metric" to logloss. The parameters were updated in an incremental way, where the model that was trained in database N was updated in database $N+1$. In the FDART, the booster was set to "dart," the objective to "binary:logistic," the "eval_metric" to logloss, and the dropout rate to 0.1 and 0.2, respectively. Regarding the FHBF algorithm, the hybrid loss scale was set to 0.1 (same as the dropout rate), the number of rounds to 20 (for evaluation purposes), and the booster to the "HFDART," which corresponds to the FDART (with the "dart" booster) but with the customized hybrid loss. According to Table 1, the FHBF yielded similar or better performance in both experimental phases 1 and 2 against the FGBT and the FDART. Even in cases where the FDART with rd = 0.1 yielded poor performance (e.g., in cases 1, 5, 7), the FHBF yielded improved performance (sensitivity 0.938, specificity 0.732 in case 1; sensitivity 0.786, specificity 0.714 in case 5; sensitivity 0.833, specificity 0.659 in case 7). In other cases, the performance was similar (e.g., in cases 4, 8). It is notable that the increased performance of the FHBF is preserved in experimental phase 2 where the class imbalance was even higher, where in case 5 the FGBT weight update process is affected by the ratio yielding poor specificity. The same occurs in case 7 regarding the FDART with rd = 0.1, where the FHBF manages to prevent the weight update process from yielding zero or infinite weights.

The increased performance of the FHBF is also confirmed by the increased AUC in the ROC curves, which are depicted in Figures 3 and 4.

The distribution of the average log loss during the training and testing procedures across cases 1–4 and cases 5–8 is depicted in Figure 5A and in Figure 5C, respectively.

In both phases, the FHBF had the lowest average training loss across cases 1–8, which highlights its resilience against overfitting. To have a concrete view of the overall loss distribution, the training and testing loss was extracted by each database and averaged across the cases in experimental phase 1 (Figure 5B) and experimental phase 2 (Figure 5D). Once more, the average loss of the FHBF was either lower or similar to the FGBT and FDART. The increased loss that appears in the "UoB," "UOI," "MHH," and "UBO" databases from phase 1 is leveraged by the FDART and FHBF (Figure 4B). The same occurs for databases "UNIVAQ," and "UOI" in phase 2 (Figure 4D).

Feature engineering

Although SHAP analysis does not currently support the "dart" booster,[33] we applied the FHBF algorithm on the best case from experimental phase 2 using the HFGBT as a booster to obtain explainable outcomes and evaluate their clinical impact.

More specifically, the FHBF provides (1) global importance plots, where the global importance of each feature is expressed as the mean absolute value for that feature over all the given samples (Figure 6A), (2) heatmaps to display the population substructure of a database where data points are clustered by their explanations and not by the original feature values (Figure 6B), (3) violin plots that display the distribution of importance for each variable (Figure 6C), and (4) waterfall plots that display explanations for individual predictions (Figure 6D). The bottom of a waterfall plot starts as the expected value of the model output, and then each row shows how the positive (red) or negative (blue) contribution of each feature moves the value from the expected model output over the background database to the model output for this prediction. According to Figure 6, all identified risk factors are in complete line with literature findings reported in a previous study.[14]

Execution time

According to Figure 7A, the average execution time of the FHBF was comparable with the FDART (and lower than the FGBT) although the total execution time (Figure 7B) of the FHBF is

Table 1. Performance evaluation results in experimental phases 1 and 2

Experimental phase	Case	Algorithm	Accuracy	Sensitivity	Specificity	AUC
Phase 1	1	FGBT	0.689	0.625	0.693	0.679
		FDART (*rd* = 0.1)	0.547	0.875	0.529	0.768
		FDART (*rd* = 0.2)	0.693	0.625	0.696	0.745
		FHBF	0.743	0.938	0.732	0.871
	2	FGBT	0.645	0.875	0.632	0.774
		FDART (*rd* = 0.1)	0.649	0.750	0.643	0.737
		FDART (*rd* = 0.2)	0.645	0.875	0.632	0.846
		FHBF	0.743	0.938	0.732	0.892
	3	FGBT	0.656	0.750	0.651	0.778
		FDART (*rd* = 0.1)	0.631	0.750	0.624	0.727
		FDART (*rd* = 0.2)	0.707	1.000	0.691	0.869
		FHBF	0.707	0.875	0.698	0.885
	4	FGBT	0.637	0.750	0.631	0.829
		FDART (*rd* = 0.1)	0.745	0.875	0.738	0.839
		FDART (*rd* = 0.2)	0.656	1.000	0.638	0.839
		FHBF	0.758	0.875	0.752	0.916
Phase 2	5	FGBT	0.497	0.857	0.479	0.693
		FDART (*rd* = 0.1)	0.599	0.643	0.596	0.737
		FDART (*rd* = 0.2)	0.667	0.786	0.661	0.796
		FHBF	0.718	0.786	0.714	0.831
	6	FGBT	0.638	0.625	0.639	0.705
		FDART (*rd* = 0.1)	0.733	0.750	0.731	0.746
		FDART (*rd* = 0.2)	0.724	0.750	0.722	0.757
		FHBF	0.750	0.875	0.741	0.786
	7	FGBT	0.682	0.667	0.683	0.804
		FDART (*rd* = 0.1)	0.565	0.833	0.555	0.855
		FDART (*rd* = 0.2)	0.671	0.667	0.671	0.778
		FHBF	0.665	0.833	0.659	0.812
	8	FGBT	0.545	1.000	0.530	0.827
		FDART (*rd* = 0.1)	0.649	1.000	0.638	0.817
		FDART (*rd* = 0.2)	0.630	1.000	0.617	0.913
		FHBF	0.675	1.000	0.664	0.914

The algorithm with the best classification performance is in bold.

directly affected by the number of trees in the forest. More specifically, the execution time was 59.11 s for 20 trees, 143.09 s for 50 trees, 315.73 s for 100 trees, 430.16 s for 150 trees, and 499.43 s for 200 trees. A detailed distribution of the time execution is also presented in Figure 6C.

DISCUSSION

In this work, we present the FHBF algorithm to address critical overfitting effects during the training of supervised learning classifiers across federated databases with increased class imbalance. To do so, a scaling parameter was first defined to adjust the shape of a hybrid loss function (based on the predefined dropout rate) to avoid weight overfitting during the error reduction (boosting) process. Confound-based random downsampling with replacement was applied to yield 1:1 matched control and target populations in each federated database. The downsampling process was repeated multiple times to avoid biases yielding an aggregated HFGBT model on each iteration. The HFGBTs from all downsampling iterations were then collected to formulate clusters of trees, where the weak clusters (i.e., those with log loss score less than the average) were discarded from the decision-making process to enhance the classification performance. Explainability analysis was finally applied based on the SHAP approach to yield explainable outcomes.

The FHBF algorithm is highly scalable since it can support both FGBT and FDART (using the xgboost "gbtree" and "dart" boosters) as base learners. In addition, the hybrid loss function can be used as an alternative to the existing loss functions that are used for binary classification tasks and are reported in the xgboost documentation,[26] such as "logistic," "logitraw," and "hinge."[26] The proposed hybrid loss function can also be used in interpretable ML algorithms toward the

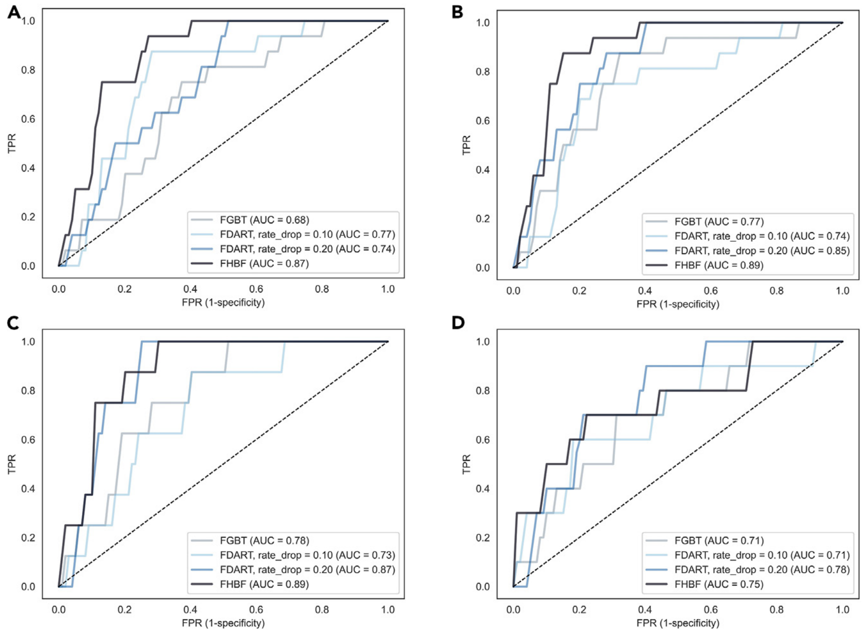

Figure 3. Receiver-operating characteristic (ROC) curves in experimental phase 1

ROC curves for the FHBF, FGBT, and FDART (with $rd \in [0.1, 0.2]$) across cases 1–4 in experimental phase 1 (A–D).

(A) Case 1. (B) Case 2. (C) Case 3. (D) Case 4.

detection of complex relationships between variables, such as the greedy decision forest.[34] The selection of the hybrid loss function is highly recommended to avoid overfitting effects that are introduced by the arbitrary definition of the dropouts. The dominance of the FHBF algorithm was demonstrated in two experimental phases involving the development of computationally complex AI models for lymphoma classification across complex data with increased class imbalance. In the second experimental phase, the class imbalance was even higher to stress the performance of the algorithms. To this end, and by taking into consideration the results from the two experimental phases, the FHBF algorithm showed the lowest average log loss distribution in the training and testing across all cases (Figure 5), as well as the best classification performance (Figures 3 and 4), where the FDART with dropout rate 0.2 achieved the second-best performance in cases 1–8 (Table 1; Figure 4). On the other hand, in cases 1, 5, and 7 (Table 1; Figures 3, 4, and 5), FGBT and FDART had poor performance compared with FHBF. Moreover, cases 5 and 7 (Table 1; Figures 3, 4, and 5) demonstrated how FHBF deals with overfitting effects against FDART and FGBT implementations. In these cases, the existing state of the art implementations yielded specificity values close to 0.5, which is an indicator of biases during the weight update process. Through its straightforward decision layer, FHBF was able to eliminate these "bad" clusters with the biased trees and thus prevented them from neglecting the model's performance. Although the federated AI model deployment time of the FHBF algorithm was larger than the FGBT and the FDART schemas, without any significant differences, the number of rounds can be reduced to leverage the computational complexity.

According to Table 2, the federated implementations of the conventional supervised learning algorithms, which rely on stochastic gradient descent (SGD), are easy to be implemented and deployed in federated environments but they are prone to overfitting effects since they suffer by linearity assumptions (i.e., the data can be explained by linear relations of the features) and thus fail to capture complex data structures (i.e., the weights of the model tend to zero or infinity). The same stands for similar approaches such as logistic regression and the federated multi-layer perceptron, which are based on linear functions. On the other hand, overfitting is less likely to occur in the federated multinomial naive Bayes (FMNB) algorithm since its hypothesis regarding the feature independence is strong. However, this assumption makes FMNB more biased and less flexible and thus fails to capture complex data structures. Contrarily, more advanced algorithms like the federated GBT methods have been proposed, which combine sequentially connected weak tree learners (in the form of an ensemble) to create a strong learner, where each tree in the ensemble minimizes the prediction error of the previous tree. However, these methods tend to be biased since the trees that are added early in the ensemble have higher impact in the decision-making process than those added earlier.

The FGBT with dropouts solves this by introducing a dropout rate that accounts for an additional set, "dropped" trees, in the decision-making process. An arbitrary definition of this rate, however, can neglect the model's performance since a higher rate will include weak trees in the decision and, thus, lower the performance of the model by causing overfitting effects, whereas a low rate might not yield any positive impact in the model's performance. To control this effect, FHBF utilizes a hybrid loss function with a scalable topology that can be adjusted according to the dropout rate to reduce overfitting. In addition, FHBF accounts for biases during the downsampling process by introducing a separation layer and a decision layer, where the former collects multiple instances of HFDARTs and drops instances with reduced score, whereas the decision layer includes only the "survivors" in the decision-making process. Although the performance of FHBF was higher in all cases, the execution time was higher, which, however, can be leveraged by reducing the number of rounds. In each round, the computational complexity was similar to the FDART and FGBT algorithms.

FHBF can be easily integrated in federated learning frameworks through a typical Python environment requiring no more than conventional libraries, such as "numpy," "scipy," "xgboost," and "pandas." The fact that the algorithm was tested in a federated AI model deployment engine, which was built under the open-source Nextcloud infrastructure

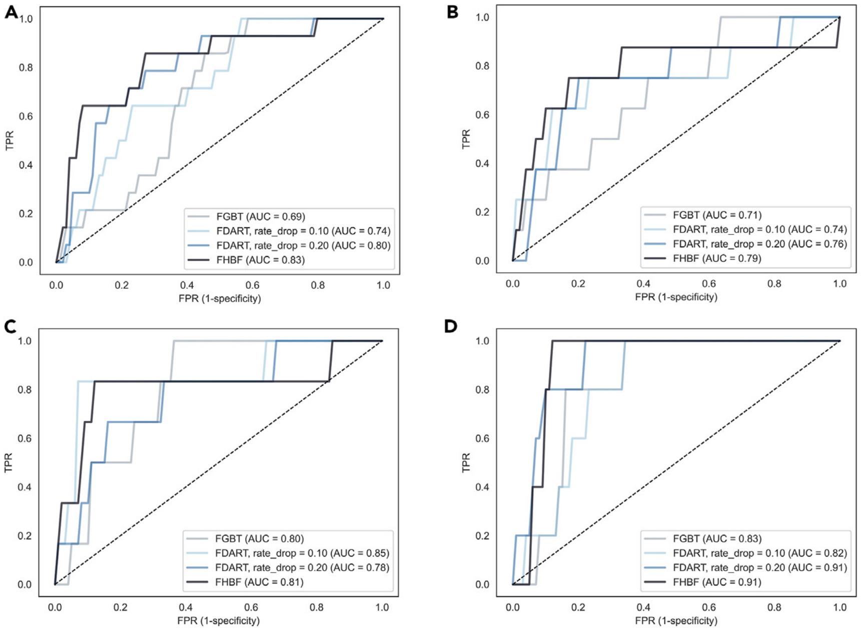

Figure 4. ROC curves in experimental phase 2

ROC curves for FHBF, FGBT, and FDART (with $rd \in [0.1, 0.2]$) across cases 5–8 from the experimental phase 2 (A–D).

(A) Case 5. (B) Case 6. (C) Case 7. (D) Case 8.

supporting the WebDAV (as defined in RFC 4918 by a working group of the Internet Engineering Task Force) API,[35] makes it compatible with Python and simplifies its integration to similar frameworks. To demonstrate the explainability and the clinical relevance of the model outcomes, we used HFGBTs as learners in the FHBF algorithm, instead of HFDARTs, to overcome the fact that the SHAP package[33] does not support the "dart" type of booster. In this case, FHBF yielded explainable outcomes that are in line with similar findings in the literature.[14]

In this work, we present the FHBF algorithm as a new paradigm toward the design, development, and deployment of robust and unbiased supervised ML models across federated databases with highly imbalanced clinical data structures, where the arbitrary selection of dropout rates combined with the increased class imbalance, can cause overfitting effects. To this end, we first defined a hybrid loss with a configurable topology that accounts for overfitting effects. The customized loss function was then introduced into the FDART schema by estimating the gradient and hessian vectors. Confound-based random downsampling with replacement was applied on each federated database to match the target population with controls based on three confounds (age, gender, and disease duration). The process was repeated K times, where the HFGBTs from each round are assembled to formulate a cluster of trees in the form of a forest. The log loss score was computed for each cluster of HFGBTs to identify and discard "weak" clusters, where the final decision-making process was based on a majority voting schema based on the predictions of the trees across the most dominant clusters. FHBF dominated in the existing state-of-the-art federated learning schemas, both in terms of classification accuracy and reduced log loss during training, and testing under two experimental cases involving the development of six federated AI models for the classification of rare lymphoma types across a pan-European data hub with rare autoimmune diseases with increased imbalance.

EXPERIMENTAL PROCEDURES

Resource availability

Lead contact

Further information and requests for data should be directed to and will be fulfilled by the lead contact, Prof. Dimitrios I. Fotiadis (fotiadis@uoi.gr).

Materials availability

The study did not generate new unique reagents.

Data and code availability

The datasets during and/or analyzed during this study are available from the corresponding author on request. The data used in the analysis were acquired by the European Union's Horizon 2020 research and innovation program under grant agreement no. 731944 and from the Swiss State Secretariat for Education, Research and Innovation SERI under grant agreement 16.0210 (HarmonicSS–HARMONIzation and integrative analysis of regional, national, and international cohorts on pSS toward improved stratification, treatment, and health policy making). Access to the HarmonicSS federated databases was obtained by signing data processor agreements between the clinical centers participating in the HarmonicSS consortium[14] and the PRECIOUS cloud infrastructure (which belongs to the University of Ioannina).

Methods

Rationale

Federated learning lies on the additive adjustment of a single estimator across multiple data structures.[8,14–16] Given a set of M-federated databases, say $\boldsymbol{D_1}, \boldsymbol{D_2}, \ldots, \boldsymbol{D_M}$, we train a ML algorithm on database $\boldsymbol{D_1}$, yielding an ML model, say $\boldsymbol{ML_{D_1}}$, and then update the model according to[2]:

$$F(x) = F(x-1) + \beta h(x), \qquad \text{(Equation 1)}$$

where $F(x)$ corresponds to the estimated mapper that is trained on database $\boldsymbol{D_i}$, $F(x-1)$ corresponds to the estimated mapper that was trained on database $\boldsymbol{D_{i-1}}$, where $i \le M$, and $\beta h(x)$ is the learner function on $\boldsymbol{D_i}$. To achieve this, we update the weights of the estimator through the SGD method which seeks for a loss function, $h(F(x_i), y_i)$, that minimizes[13]:

$$L(\boldsymbol{w}) = argmin\left(\frac{1}{N}\sum_{i=1}^{N} h(F(x_i), y_i) + ar(\boldsymbol{w})\right), \qquad \text{(Equation 2)}$$

where, x_i is the ith instance, y_i is the target, $\boldsymbol{w}$ is a weight vector, $h(.)$ is a loss function, a is a hyperparameter, $r(\boldsymbol{w})$ is a regularizer, $L(.)$ is the objective, and $F(x_i)$ is a linear score function. Solving Equation 2 yields the weight update formula:

$$w_i = w_{i-1} - \eta_t(\nabla_w h(F(x_i), y_i) + a\nabla_w r(w)), \qquad \text{(Equation 3)}$$

where i is the stage, w_{i-1} is the weight estimation at stage $i-1$, η_t is a non-negative learning rate parameter, and $\nabla_w h(F(\boldsymbol{x_i}), \boldsymbol{y_i})$ is the gradient of the loss function $h(.)$. A typical pseudocode that summarizes the backbone of federated learning is presented in Algorithm 1. An ML algorithm is trained on the first database yielding the initial weights which are additively updated across the rest of the training databases through Equation 3.

Existing federated learning implementations

Federated regressors and SVM. By replacing the loss function in Equation 3 we can obtain federated implementations of conventional SGD-based

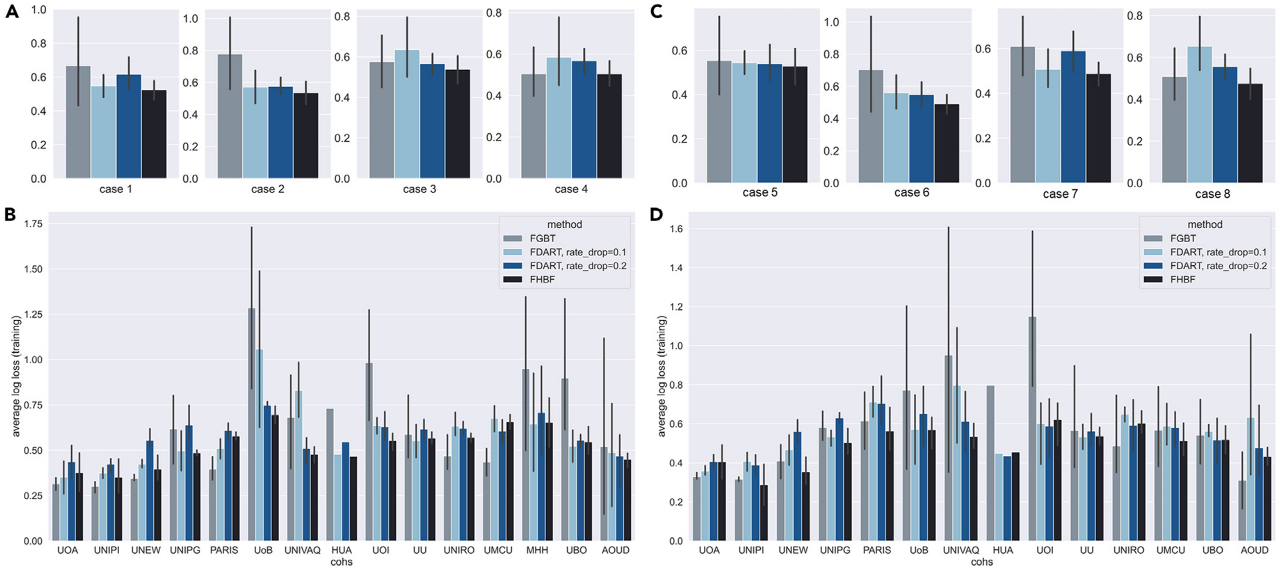

Figure 5. Average loss distributions

Average training and testing loss distribution across (A) cases 1–4 from experimental phase 1, (B) training and testing databases in cases 1–4 from experimental phase 1, (C) cases 5–8 from experimental phase 2, and (D) training and testing databases involved in cases 5–8 from experimental phase 2. The error bars refer to the 95% confidence intervals of the estimated distributions using bootstrapping.

classifiers. For example, if we replace the function $h(F(x_i), y_i)$: (1) with a logistic loss function $ln\,(1 + exp\,(-\,y_i f(x_i)))$, we can obtain the federated logistic regression classifier; (2) with the hinge loss function $max\,(0, 1 - y_i f(x_i))$, we can obtain the federated SVM classifier; and (3) with the perceptron loss $max\,(0, -y_i f(x_i))$, we can obtain the federated perceptron classifier. The regularization function, $r(w)$, in Equation 3 can be also replaced to derive either an $l1$- or $l2$-norm regularization, or even the elastic-net regularization for a combination of both.

FMNB. The multinomial naive Bayes classifier is preferred in the case where the features are discrete.[22] Given an N-dimensional input vector,

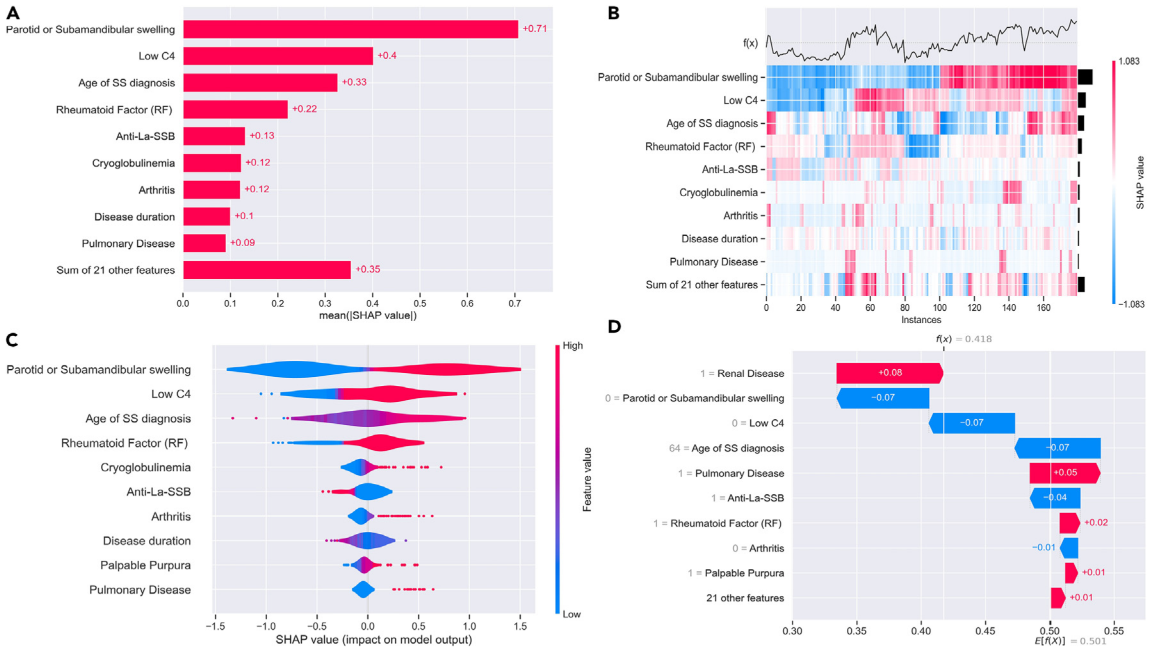

Figure 6. SHAP analysis results on a randomly selected case from experimental phase 2

(A) Global importance of each feature.

(B) The population substructure is clustered by their explanations.

(C) Distribution of importance for each variable.

(D Explanations for individual predictions.

CellPress
OPEN ACCESS

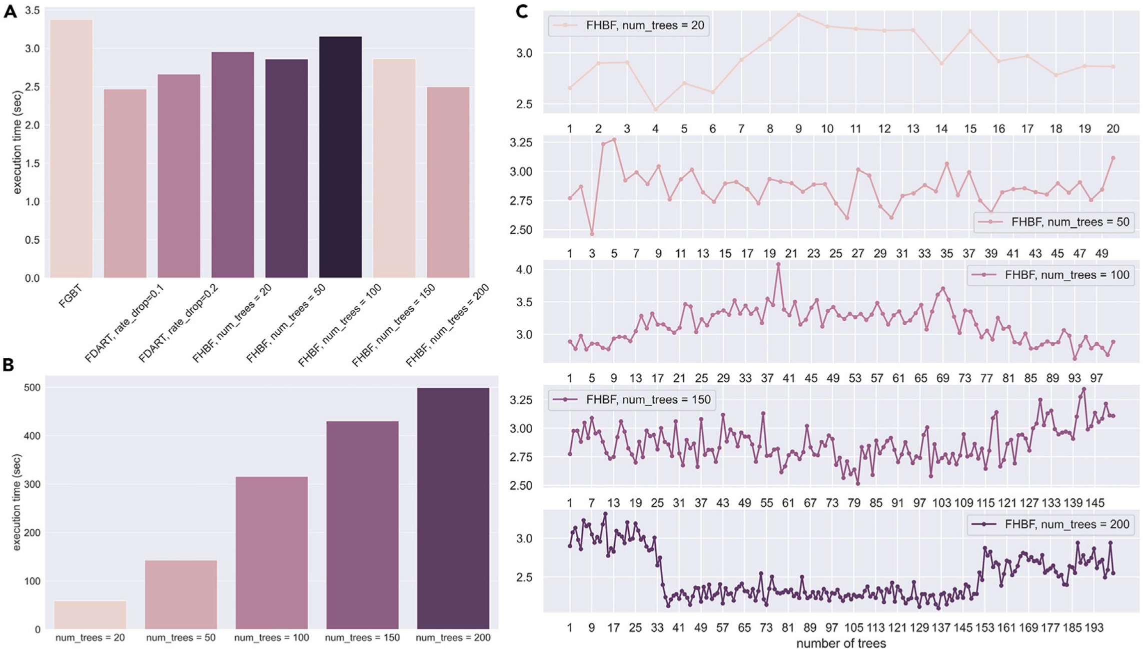

Figure 7. Computational performance in terms of execution time (s)

(A) The execution time (s) of FGBT and FDART and the average execution time (s) for FHBF with 20, 50, 100, 150, and 200 trees.

(B) The total execution time (s) of FHBF with 20, 50, 100, 150, and 200 trees.

(C) The distribution of the individual execution times (s) of FHBF with 20, 50, 100, 150, and 200 trees.

assume $x = (x_1, x_2, \ldots, x_N)$, where each x_i is the frequency of an event t_i, the probability that x belongs to the class, assume c_k, is given by the multinomial distribution. The maximum *a*-posterior (MAP) class is derived by the logarithmic expression of the multinomial distribution[23] which can then be solved as a linear equation:

$$c_{MAP} = argmax_{c_k}\left[log(P(c_k)) + \sum_{i=1}^{N} log(P(t_i|c_k))\right], \quad \text{(Equation 4)}$$

where $P(c_k)$ is the likelihood of class c_k and $P(t_i|c_k)$ is the conditional probability of t_i occurring in c_k.

FGBT. The GBT algorithm introduces a boosting stage to sequentially optimize a differentiable error loss function.[24,25] In this work, we first define the FGBT algorithm which seeks for a weak learner, say $f_i(x)$, at step i so that:

$$F_i(x) = F_{i-1}(x) + \gamma_i f_i(x), \quad \text{(Equation 5)}$$

where $F_{i-1}(x)$ is the ensemble at step $i-1$, a_i is the weight that is given to the classifier at step i, $h_i(x)$ is the outcome of the weak classifier at step i, $f_i(x)$ is a weak learner and γ_i is defined according to the SGD approach as in:

$$\gamma_i = argmin_{\gamma}\left(\sum_{j=1}^{n} h(y_j, F_{i-1}(x_j) - \gamma\nabla_{F_{i-1}} h(y_j, F_{i-1}(x_j))\right), \quad \text{(Equation 6)}$$

where $h(.)$ is the error loss function and n is the number of samples. In this work, we extended the XGBoost algorithm with the "gbtree" booster[26] to facilitate the weight update across federated databases.

FDART. A critical issue in FGBT is the fact that the algorithm combines regression trees with a small learning rate and thus trees that are added early in the ensemble are more significant than those added late. To this end, the DART booster has been proposed in the literature,[20] which scales (combines) the dropped trees with the new tree, on each round, by an appropriate factor, which ensures that their combination will have the same effect on the outcome. To do so, the DART is trained on intermediate instances of the random subset that is selected by GBT and thus prevents the construction of trivial trees. For a model, say M, with $M(t)$ denoting the prediction for point t, DART creates a subset[16]:

$$\{(t, -\nabla_t L(T(t)))\}, \quad \text{(Equation 7)}$$

where $\nabla L(T(t))$ is the gradient of the loss function $L(T(t))$. Thus, a new label with values $-\nabla_t L(T(t))$ is created for each sample t in the training database. In this work, we extended the XGBoost algorithm with the "dart" booster[26] to facilitate the weight update across federated databases.

Addressing open challenges in FGBT and FDART implementations: The FHBF architecture

According to Figure 8, the FHBF architecture is comprised of three individual layers: (1) the weight update layer, (2) the separation layer, and (3) the decision-making layer. In the weight update layer, the FHBF algorithm is utilized and recursively applied across the federated databases. The weight update process is repeated K tmes by applying random downsampling with replacement with respect to a set of pre-defined confound factors among the control group and the target group in each federated database. The weight update process is orchestrated by the central node (CN), which communicates with the federated AI model handler and the federated AI model collector. The former is responsible for the transmission and storage of the individual model weights from one database to another, whereas the federated AI model collector is responsible for gathering the individual HFDART models to formulate a set of clusters with the HFDARTs from each round. This set is referred to as a forest of HFDARTs. In the separation layer, the weak HFDARTs models in the forest are identified by a log loss score and are eliminated. The remaining HFDARTs are used for the final decision-making based on majority voting. The output stage includes the final predictions along with explainable AI scores.

FHBF information flow

The information flow that reflects the core operations in FHBF is depicted in Figure 9. According to Figure 9, the FHBF core parameters, including the number of rounds (K), the number of training databases (N), and the number of testing databases (M), are first defined. In each round, say

Table 2. Comparison with federated implementations of existing supervised learning algorithms

Algorithm	Advantages	Weaknesses
Federated SGD-based (e.g., support vector machines, logistic regression)	easy to be implemented and deployed in federated environments, low computational complexity	poor classification performance, prone to overfitting during federated training
FMNB	easy to be implemented and deployed in federated environments, low computational complexity, immune against overfitting	probabilistic approach, biases are introduced in the results from several assumptions regarding the independence of the set of input features
FGBT	favorable classification performance due to the error reduction through boosting, easy deployment in federated environments, scalable, starts pruning trees backward based on the depth-first approach	low to medium computational complexity especially with increasing number of boosting rounds
FDART	favorable classification performance due to the error reduction through boosting, easy deployment in federated environments, scalable, allows for the use of dropout rates which can significantly enhance the performance	low to medium computational complexity especially with increasing number of boosting stages, arbitrarily defined dropout rates can lead to overfitting and neglect the performance of the model
FHBF	increased classification performance, ideal in federated cases with increased class imbalance, allows for the use of dropout rates that can significantly enhance the performance, adjusted hybrid loss topology, which avoids overfitting considering the dropout rate	medium to high computational complexity, which can be affected by the number of iterations

FDART, federated gradient boosting trees with dropout rate; FGBT, federated gradient boosting trees; FHBF, federated hybrid boosted forests; FMNB, federated multinomial naive Bayes; SGD, stochastic gradient descent.

$j \in [1, K]$, the algorithm gets sequential access to each training database, say $i \in [1, N]$, in the federated system. Random downsampling with replacement is applied on the training database to match the target group with the control group according to a predefined downsampling ratio (usually 1:1). The matching process is applied with respect to confound factors, such as age, gender, and disease duration, to avoid biases during population matching. The hybrid loss function is then defined by the scaling parameter with respect to the dropout rate. The first- and the second-order gradient of the loss function are then computed and utilized for the weight update process. The updated weights are entered in a boosting process consisting of k rounds. In each boosting round, the weights of the model are updated to minimize the prediction loss.

When the boosting process ends, the CN is invoked, and the weights of the model are stored. These weights are used for the training process in the next training database until all training databases participate in the analysis (i.e., until $j = N$). Once the training process is terminated, a cluster of federated models is created (i.e., an FHBF). Each cluster is evaluated in each testing database, say $m \in [1, M]$, and the binary cross-entropy loss (or log loss) is then estimated for each cluster. As soon as the testing process is terminated, the weak clusters (i.e., those with log loss score less than the average log loss of the forest) are eliminated from the final decision-making process. Shapley additive explanation analysis is finally applied on the strong clusters to derive explainable scores for each input feature that participated in the workflow with respect to the target outcome.

Weight update layer

Hybrid loss function. A main problem in the FDART algorithm is to account for overfitting effects in the selection of the dropout rate, say r. The fact that the algorithm combines many regression trees with a small learning rate and thus trees that are added early in the ensemble are more significant than trees added late. To solve this we propose a hybrid loss function that combines the *logcosh* loss,[27] say f, with the Huber loss,[27] say g, where the topology of the loss function is controlled by a parameter δ value. The *logcosh* loss f is defined as:

$$f(y, \hat{y}) = log\,(cosh(y - \hat{y})), \quad \text{(Equation 8)}$$

where y is the target vector and $\hat{y}$ is the vector with the estimations. On the other hand, the modified Huber loss, say $g(y, \hat{y}, \delta)$ is defined as:

$$g(y, \hat{y}, \delta) = \begin{cases} \frac{1}{2}|y - \hat{y}|^2, |y - \hat{y}| \leq \delta \\ \delta\left(|y - \hat{y}| - \frac{1}{2}\delta\right), |y - \hat{y}| > \delta \end{cases}, \quad \text{(Equation 9)}$$

where δ is a scaling parameter in the Huber loss. Then, f and g are combined into a hybrid loss function, say $h = f * g$, which is calculated based on the product rule yielding the following first-order gradient:

$$\nabla h = log\,(cosh(y - \hat{y}))((y - \hat{y}) / \sqrt{s}) + g(y, \hat{y}, \delta) tanh(y - \hat{y}) \quad \text{(Equation 10)}$$

and the second-order gradient:

$$\nabla^2 h = \left(\frac{1}{cosh^2(y - \hat{y})}\right) g(y, \hat{y}, \delta) + 2\left(tanh(y - \hat{y})\left(\frac{y - \hat{y}}{\sqrt{s}}\right)\right) + tanh(y - \hat{y})\left(\frac{1}{s\sqrt{s}}\right) \quad \text{(Equation 11)}$$

where s is an approximation factor defined as $1 + ((y - \hat{y})/\delta)^2$.[28] The dropout rate rd was finally set equal to the scaling parameter δ so that the shape of the loss function would be steeper around 0 to avoid weight overfitting for large r.

Weight update function. The rationale of the gradient boosting process lies on the transformation of a set of weak learners into a much a stronger one by additively updating the weights of the model until the prediction error is minimized. The error minimization process is usually based on the stochastic gradient approach (SGD). Given a set of N observations $\{(\boldsymbol{x_1}, y_1), (\boldsymbol{x_2}, y_2), \ldots, (\boldsymbol{x_N}, y_N)\}$, where $\boldsymbol{x_i} \in \boldsymbol{R^N}$, the objective is to obtain an estimated function, $\tilde{F}(\boldsymbol{x})$, mapping $\boldsymbol{x}$ to y, which minimizes the expected value of a loss function, assume $L(y, F(\boldsymbol{x}))$. The gradient boosting process incrementally seeks for estimations of a mapper at a stage $m \in M$, assume $F_m(x)$, which can be analytically expressed as follows:

$$F_i(x) = F_{i-1}(x) + \gamma_i f_i(x) F_i(x) = F_{i-1}(x) - \gamma_i \sum_{j=1}^{n} \nabla_{F_{i-1}} L(y_j, F_{i-1}(x_j)), \quad \text{(Equation 12)}$$

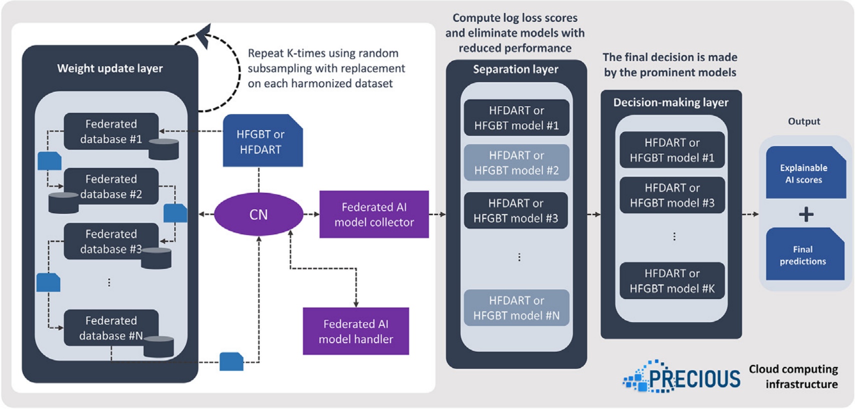

Figure 8. An illustration of the FHBF architecture

where the regularization objective is approximated according to Taylor's theorem[24] as in:

$$E(t) \approx \sum_{i=1}^{N} \left[L\left(y_i, \tilde{y}_{i,t-1}\right) + g_i f_t(x_i) + \frac{1}{2} h_i f_t^2(x_i) \right] + r, \quad \text{(Equation 13)}$$

where $l(.)$ is the loss function at step t, $\tilde{y}_{i,t-1}$ is the estimated target at step $t-1$, y_i is the real target, and r is a regularization function:

$$r = \gamma L + \frac{1}{2} \lambda \sum_{j=1}^{L} w_j^2, \quad \text{(Equation 14)}$$

where w is the weight on the leaves, γ is a constant value, and L is the total number of leaves in each tree. Here, the first- and second-order gradients are introduced in Equation 13, yielding the FHBF regularization objective:

$$E(t) \approx \sum_{i=1}^{N} \left[l\left(y_i, \tilde{y}_{i,t-1}\right) + \left(log\left(cosh(y - \hat{y})\right)\right) \times \left(\frac{y - \hat{y}}{\sqrt{s}}\right) + g(y, \hat{y}, \delta) tanh(y - \hat{y}) \right] + r. \quad \text{(Equation 15)}$$

Confound-based class imbalance handling. A crucial challenge that is introduced during the training across federated databases is the increased class imbalance among the control and the target groups in each database. To solve this, random downsampling with replacement was applied to extract a balanced set of training instances in each database. The downsampling process is based on a pre-defined ratio, say dr, which determines the population size of the control group with respect to the target group. The downsampling process was applied on each database separately, and was finally repeated K tmes to obtain an unbiased estimation of the model performance. In each iteration, confound factors were taken into consideration to ensure subgroup matching without statistically significant differences. More specifically, given a set of N-profound factors (features), say $\{\mathbf{x_1}, \mathbf{x_2}, \ldots, \mathbf{x_N}\}$, we seek for a random control subgroup where the patients' clinical profiles do not statistically deviate from those in the target group. To do so, the non-parametric Wilcoxon rank-sum test (or Student's t test in the case of normality upon a Shapiro-Wilk test) was applied on the continuous confound factors and the chi-squared test/Fisher's exact test in the case of the discrete factors to evaluate whether the target subgroup and a randomly selected control subgroup, in each database, does not significantly deviate at a 95% confidence level.

Separation layer

Assembly stage and scoring procedure. In the assembly stage, the K individual HFGBT models are collected to formulate a set of HFGBTs in the form of a forest, say C, as in:

$$C = \{HFGBT_1, HFGBT_2, \ldots, HFGBT_K\}, \quad \text{(Equation 16)}$$

where $HFGBT_i$ corresponds to the HFGBT from the ith federated training round. In the case where the HFDART is used as a booster, then Equation 16 is updated accordingly.

The binary cross-entropy (log loss) score is estimated for each model in the forest C as in:

$$H(C_j) = -\frac{1}{N} \sum_{i=1}^{N} y_i \, log(p(y_i)) + (1 - y_i) log\,(1 - p(y_i)), \quad \text{(Equation 17)}$$

where C_j corresponds to the model $HFGBT_j$, $j = 1, \ldots, K$, y_i is the target of the ith instance in the testing database, and $p(y_i)$ is the probability of the target class.

Collecting the final "survivors". Clusters of HFGBTs (or HFDARTs in the case where HFDART is used as a booster in FHBF) with log loss score below the average log loss score in the forest C are marked as weak candidates and are discarded from the final decision-making process.

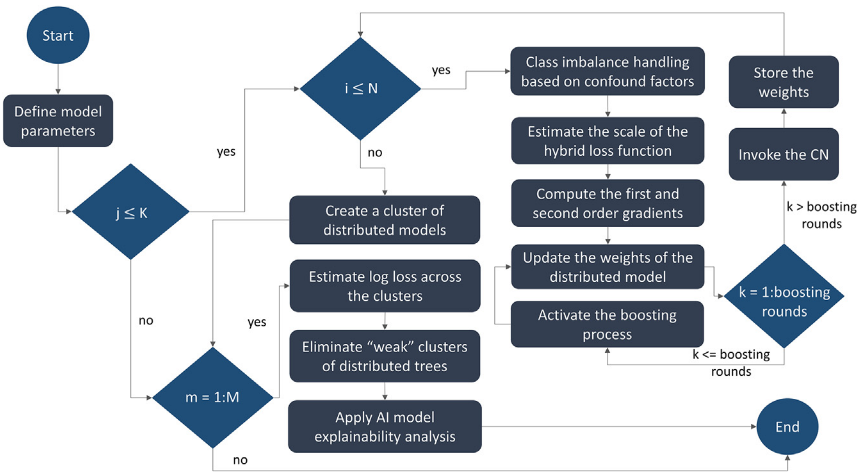

Figure 9. An illustration of the FHBF information flow

Algorithm 1. A pseudocode for federated learning

def federated_learning(F ,$\boldsymbol{D} = \{\boldsymbol{D_0}, \boldsymbol{D_1}, \boldsymbol{D_2} \ldots, \boldsymbol{D_M}\}, \boldsymbol{w_0}$):
fit an estimator $\boldsymbol{F_o}$ on the database $\boldsymbol{D_o}$ yielding $\boldsymbol{w_0}$
for $i = 0{:}M$ do:
  retrieve weight vector w_{i-1} from the previous execution
  solve $w_i = w_{i-1} - \eta_t(\nabla_w h(F(x_i), y_i) + a\nabla_w r(w))$
  update (3)
return $[w_i, F_i]$

Decision-making layer

As a final step, majority voting is applied on the “survivors” to derive the final predictions from a testing database. To this end, the decision-making process is formulated as follows:

$$\tilde{y}_i = \begin{cases} 1, if \sum_{j=1}^{K} \tilde{y}_{i,j} > K/2 \\ 0, o.w. \end{cases}, \quad \text{(Equation 18)}$$

where $\tilde{y}_i$ is the predicted value for the ith instance. Weighted voting is also supported.

Explainability. The SHAP method[29,30] was used to provide explainable outcomes using the HFGBT booster. The SHAP value for feature x_j is defined as:

$$\varphi_j(v) = \sum_{T \subseteq x \backslash \{x_j\}} \frac{|T|!(n - |T| - 1)!}{n!}(v(T \cup \{x_j\}) - v(T)), \quad \text{(Equation 19)}$$

where T is a subset of a set of n-input features, say $\{x_1, x_2, \ldots, x_n\}$, $v(T)$ is a vector of the classification outcomes given the features in T, and $v(T \cup \{x_j\})$ is a vector of the classification outcomes.

The FHBF pseudocode

A pseudocode of the FHBF is presented in Algorithm 2. The input parameters of the FHBF include (1) “**K**,” which refers to the number of HFGBTs or HFDARTs in the forest, (2) “**N**,” which refers to the number of training databases, (3) “**M**,” which refers to the number of testing database(s), (4) “**train_databases**,” which refers to the locations of the training databases for the WebDAV API function, (5) “**test_databases**, which refers to the locations of the testing databases for the WebDAV API function, (6) “**matching**,” which refers to whether the user wants to apply population matching or not during the downsampling phase in each database, (7) “**booster**,” which refers to the type of booster (either HFGBT or HFDART), (8) “**rate_drop**,” which refers to the dropout rate, (9) “**delta**,” which refers to the scale of the hybrid loss (by default equal to “rate_drop”), (10) “**loss_score**,” which refers to the scoring function, and (11) “**voting**,” which refers to the voting approach (majority or weighted). Then, the FHBF estimates the weight of the model in the first federated database and stores them in the CN. The scaling parameter is determined along with the first- and second-order gradients based on Equations 10 and 11. The weights of the model are sequentially updated according to Equation 15 and stored in the computing node. The final model, say M_N, is then retrieved and evaluated on a set of one or more testing databases. The weights of M_N and the predictions are stored in cluster C_j. The process is repeated K times and the collected clusters are aggregated into the forest C. Majority voting is applied to derive the final predictions y which are returned in the output.

Computing power and source code

The executions were performed on a CN with two Intel(R) Xeon(R) Gold 5220R (4 GHz maximum turbo frequency each), which is part of a high-performance computing infrastructure that was designed for data-intensive tasks (i.e., the PRECIOUS system in Greece). The source code of FHBF implementation and validation can be found in: https://github.com/vpz4/FHBF/tree/main.

SUPPLEMENTAL INFORMATION

Supplemental information can be found online at https://doi.org/10.1016/j.patter.2023.100893.

Algorithm 2. A pseudocode of the FHBF algorithm

Input parameters
K: number of HFGBTs or HFDARTs in the forest
N: number of training databases
M: number of testing databases
train_databases: locations of the training databases for access
test_databases: locations of the testing database(s) for access
matching: whether to apply triple confound-based downsampling or not
booster: hybrid FGBT (HFGBT) or hybrid FDART (HFDART)
rate_drop: the dropout rate
delta: scaling hyperparameter of the hybrid loss function
loss score: the scoring function that determines the percentage of dumped trees in the forest
voting: whether to apply majority voting or weighted voting on the remaining trees
def FHBF (K, M, N, train_databases, test_databases, matching, delta, loss score, voting):
for j in range(0, $\boldsymbol{K}$) do:
  for i in range(0, $\boldsymbol{N}$) do:
    determine the scaling parameter δ based on the dropout rate rd
    compute first- and second-order gradients of the hybrid loss function according to Equations 10 and 11
    update the weights of M_i on federated database $i+1$ according to Equation 15
    store the weights of the federated model M_{i+1} in the CN
  retrieve the final federated model M_N from the training stage
  evaluate the performance of M_N on the testing databases as specified in **test_databases**
  store the weights of the M_N in cluster C_j along with the predictions
estimate the **log loss** to drop the “weak” clusters in C having the highest loss
apply **voting** to derive the final predictions, say y, from the survivors in C
return y;

CellPress
OPEN ACCESS

ACKNOWLEDGMENTS

The project is co-financed by Greece and the European Union—European Regional Development Fund (ERDF) under the Operational Program "Competitiveness Entrepreneurship Innovation" (EPAnEK), NSRF 2014–2020 (project code: 82863, MIS: 5047133).

AUTHOR CONTRIBUTIONS

V.C.P. implemented the federated AI workflows and prepared Figures 1–8. F.K. configured the cloud computing infrastructure and the federated databases. T.P.E. participated in the design of the federated AI workflows and the explainability analysis. D.I.F. defined the main aspects of the federated AI workflows and orchestrated the requirements for access to the federated databases. A.G.T. defined the clinical problem. A.G. provided the population characteristics for the study design procedure. V.C.P., F.K., T.P.E., A.G., and D.I.F. wrote the main manuscript. A.G. and A.G.T. defined the clinical study design including the collection of data sharing and data processor agreements. A.G.T. and D.I.F. reviewed the manuscript and provided valuable comments for overall quality improvement.

DECLARATION OF INTERESTS

The authors declare no competing interests.

Received: August 25, 2023
Revised: November 3, 2023
Accepted: November 10, 2023
Published: December 12, 2023

REFERENCES

1. Miah, S.J., Camilleri, E., and Vu, H.Q. (2022). Big Data in healthcare research: a survey study. J. Comput. Inf. Syst. *62*, 480–492.
2. Majnarić, L.T., Babič, F., O"Sullivan, S., and Holzinger, A. (2021). AI and big data in healthcare: towards a more comprehensive research framework for multimorbidity. J. Clin. Med. *10*, 766.
3. Shilo, S., Rossman, H., and Segal, E. (2020). Axes of a revolution: challenges and promises of big data in healthcare. Nat. Med. *26*, 29–38.
4. Leonardsen, A.C.L., Hardeland, C., Helgesen, A.K., and Grøndahl, V.A. (2020). Patient experiences with technology enabled care across healthcare settings-a systematic review. BMC Health Serv. Res. *20*, 1–17.
5. Zerka, F., Barakat, S., Walsh, S., Bogowicz, M., Leijenaar, R.T., Jochems, A., and Lambin, P. (2020). Systematic review of privacy-preserving distributed machine learning from federated databases in health care. JCO clinical cancer informatics *4*, 184–200.
6. Antunes, R.S., André da Costa, C., Küderle, A., Yari, I.A., and Eskofier, B. (2022). Federated Learning for Healthcare: Systematic Review and Architecture Proposal. ACM Transactions on Intelligent Systems and Technology (TIST) *13*, 1–23.
7. Kumar, Y., and Singla, R. (2021). Federated learning systems for healthcare: perspective and recent progress. Federated Learning Systems, 141–156.
8. Xu, J., Glicksberg, B.S., Su, C., Walker, P., Bian, J., and Wang, F. (2021). Federated learning for healthcare informatics. Journal of Healthcare Informatics Research *5*, 1–19.
9. Benczúr, A.A., Kocsis, L., and Pálovics, R. (2018). Online machine learning in big data streams. Preprint at arXiv.
10. Vilalta, R., and Drissi, Y. (2002). A perspective view and survey of meta-learning. Artif. Intell. Rev. *18*, 77–95.
11. Gepperth, A., and Hammer, B. (2016). Incremental learning algorithms and applications. In European symposium on artificial neural networks (ESANN).
12. Wu, Y., Chen, Y., Wang, L., Ye, Y., Liu, Z., Guo, Y., and Fu, Y. (2019). Large scale incremental learning. In Proceedings of the IEEE/CVF Conference on Computer Vision and Pattern Recognition, pp. 374–382.
13. Bertsekas, D.P. (2011). Incremental gradient, subgradient, and proximal methods for convex optimization: A survey. Optimization for Machine Learning *2010*, 3.
14. Pezoulas, V.C., Goules, A., Kalatzis, F., Chatzis, L., Kourou, K.D., Venetsanopoulou, A., and Fotiadis, D.I. (2022). Addressing the clinical unmet needs in primary Sjögren"s Syndrome through the sharing, harmonization and federated analysis of 21 European cohorts. Comput. Struct. Biotechnol. J.
15. Li, Q., Wen, Z., and He, B. (2020). Practical federated gradient boosting decision trees. Proc. AAAI Conf. Artif. Intell. *34*, 4642–4649.
16. Shen, Z., Hassani, H., Kale, S., and Karbasi, A. (2022). Federated functional gradient boosting. In International Conference on Artificial Intelligence and Statistics (PMLR), pp. 7814–7840.
17. Brisimi, T.S., Chen, R., Mela, T., Olshevsky, A., Paschalidis, I.C., and Shi, W. (2018). Federated learning of predictive models from federated electronic health records. Int. J. Med. Inf. *112*, 59–67.
18. Zhang, C., Xie, Y., Bai, H., Yu, B., Li, W., and Gao, Y. (2021). A survey on federated learning. Knowl. Base Syst. *216*, 106775.
19. Pezoulas, V.C., Kalatzis, F., Exarchos, T.P., Chatzis, L., Gandolfo, S., Goules, A., and Fotiadis, D.I. (2021). A federated AI strategy for the classification of patients with Mucosa Associated Lymphoma Tissue (MALT) lymphoma across multiple harmonized cohorts. In 2021 43rd Annual International Conference of the IEEE Engineering in Medicine & Biology Society (EMBC) (IEEE), pp. 1666–1669.
20. Vinayak, R.K., and Gilad-Bachrach, R. (2015). Dart: Dropouts meet multiple additive regression trees. In Artificial Intelligence and Statistics (PMLR), pp. 489–497.
21. Hauschild, A.C., Lemanczyk, M., Matschinske, J., Frisch, T., Zolotareva, O., Holzinger, A., and Heider, D. (2022). Federated Random Forests can improve local performance of predictive models for various healthcare applications. Bioinformatics *38*, 2278–2286.
22. Marikani, T., and Shyamala, K. (2019, February). Modified Multinomial Naïve Bayes Algorithm for Heart Disease Prediction. In Intelligent Communication Technologies and Virtual Mobile Networks (Springer), pp. 294–300.
23. Berrar, D. (2018). Bayes" theorem and naive Bayes classifier. In Encyclopedia of Bioinformatics and Computational Biology, *403* (ABC of Bioinformatics).
24. Friedman, J.H. (2001). Greedy function approximation: a gradient boosting machine. Ann. Stat. 1189–1232.
25. Chen, T., and Guestrin, C. (2016). Xgboost: A scalable tree boosting system. In Proceedings of the 22nd ACM SIGKDD International Conference on Knowledge Discovery and Data Mining, pp. 785–794.
26. Wade, C., and Glynn, K. (2020). Hands-on gradient boosting with XGBoost and scikit-learn: Perform accessible machine learning and extreme gradient boosting with Python (Packt Publishing Ltd.).
27. Wang, Q., Ma, Y., Zhao, K., and Tian, Y. (2022). A comprehensive survey of loss functions in machine learning. Annals of Data Science *9*, 187–212.
28. Hastie, T., Tibshirani, R., Friedman, J.H., and Friedman, J.H. (2009). The Elements of Statistical Learning: Data Mining, Inference, and Prediction, *2* (springer), pp. 1–758.
29. Lundberg, S.M., and Lee, S.I. (2017). A unified approach to interpreting model predictions. Adv. Neural Inf. Process. Syst. *30*.
30. Sundararajan, M., and Najmi, A. (2020). The many Shapley values for model explanation. In International conference on machine learning (PMLR), pp. 9269–9278.
31. Goules, A.V., Exarchos, T.P., Fotiadis, D.I., and Tzioufas, A. (2021). The clinical and technical impact of the HarmonicSS project. Clin. Exp. Rheumatol.

32. Pedregosa, F., Varoquaux, G., Gramfort, A., Michel, V., Thirion, B., Grisel, O., and Duchesnay, É. (2011). Scikit-learn: Machine learning in Python. the Journal of machine learning research *12*, 2825–2830.

33. Bifarin, O.O. (2023). Interpretable machine learning with tree-based shapley additive explanations: Application to metabolomics datasets for binary classification. Plos one *18*, e0284315.

34. Pfeifer, B., Baniecki, H., Saranti, A., Biecek, P., and Holzinger, A. (2022). Multi-omics disease module detection with an explainable Greedy Decision Forest. Sci. Rep. *12*, 16857.

35. Cath, C. (2021). The technology we choose to create: Human rights advocacy in the Internet Engineering Task Force. Telecommun. Pol. *45*, 102144.

Patterns, Volume *5*

Supplemental information

FHBF: Federated hybrid boosted forests with dropout rates for supervised learning tasks across highly imbalanced clinical datasets

Vasileios C. Pezoulas, Fanis Kalatzis, Themis P. Exarchos, Andreas Goules, Athanasios G. Tzioufas, and Dimitrios I. Fotiadis

Supplementary Material

Supplementary Table 1. Descriptive statistics of the features participating in cases 1-8.

ID	Feature	Type	Range	Mean/Most frequent value
1	Gender	discrete	[0, 1]	1 (females)
2	Age of SS diagnosis	continuous	[10, 95]	51.59
3	Disease duration	continuous	[0, 49]	7.21
4	Dry mouth	discrete	[0, 1]	1 (presence)
5	Dry eyes	discrete	[0, 1]	1 (presence)
6	Parotid or Submandibular swelling	discrete	[0, 1]	0 (absence)
7	Raynaud”s phenomenon	discrete	[0, 1]	0 (absence)
8	Arthritis	discrete	[0, 1]	0 (absence)
9	Renal disease	discrete	[0, 1]	0 (absence)
10	Tubulointerstitial nephritis	discrete	[0, 1]	0 (absence)
11	Glomerulopathy	discrete	[0, 1]	0 (absence)
12	Pulmonary disease	discrete	[0, 1]	0 (absence)
13	Small airway disease	discrete	[0, 1]	0 (absence)
14	Interstitial lung disease	discrete	[0, 1]	0 (absence)
15	Liver disease	discrete	[0, 1]	0 (absence)
16	Autoimmune hepatitis (AIH)	discrete	[0, 1]	0 (absence)
17	Primary biliary cholangitis (PBC)	discrete	[0, 1]	0 (absence)

18	Nervous System Disease	discrete	[0, 1]	0 (absence)
19	Peripheral Nervous System Disease	discrete	[0, 1]	0 (absence)
20	Central Nervous System Disease	discrete	[0, 1]	0 (absence)
21	Palpable purpura	discrete	[0, 1]	0 (absence)
22	Muscular System Disease	discrete	[0, 1]	0 (absence)
23	Idiopathic Inflammatory Myopathy (IIM)	discrete	[0, 1]	0 (absence)
24	Inclusion Body Myositis (IBM) documented with Biopsy	discrete	[0, 1]	0 (absence)
25	Anti-La-SSB	discrete	[0, 1]	0 (absence)
26	Anti-Ro-SSA	discrete	[0, 1]	1 (presence)
27	Rheumatoid Factor (RF)	discrete	[0, 1]	1 (presence)
28	Antinuclear Antibodies (ANA)	discrete	[0, 1]	1 (presence)
29	Low C4	discrete	[0, 1]	0 (absence)
30	Cryoglobulinemia	discrete	[0, 1]	0 (absence)
31	Lymphoma	discrete	[0, 1]	0 (absence)
32	B-cell Mucosa-associated Lymphoid Tissue (MALT) Lymphoma	discrete	[0, 1]	0 (absence)
